# Environmental influences on *Aedes aegypti* catches in Biogents Sentinel traps during a Californian “rear and release” program: Implications for designing surveillance programs

**DOI:** 10.1371/journal.pntd.0008367

**Published:** 2020-06-12

**Authors:** Kyran M. Staunton, Jacob E. Crawford, Devon Cornel, Peter Yeeles, Mark Desnoyer, Josh Livni, Jodi Holeman, F. Stephen Mulligan, Nigel Snoad, Scott A. Ritchie

**Affiliations:** 1 College of Public Health, Medical and Veterinary Sciences, James Cook University, Smithfield, Australia; 2 Australian Institute of Tropical Health and Medicine, James Cook University, Smithfield, Australia; 3 Verily Life Sciences, South San Francisco, California, United States of America; 4 Consolidated Mosquito Abatement District, Parlier, California, United States of America; 5 College of Science & Engineering, James Cook University, Smithfield, Australia; Duke-NUS GMS, SINGAPORE

## Abstract

As *Aedes aegypti* continues to expand its global distribution, the diseases it vectors (dengue, Zika, chikungunya and yellow fever) are of increasing concern. Modern efforts to control this species include “rear and release” strategies where lab-reared mosquitoes are distributed throughout the landscape to replace or suppress invasive populations. These programs require intensive surveillance efforts to monitor their success, and the Biogents Sentinel (BGS) trap is one of the most effective tools for sampling adult *Ae*. *aegypti*. BGS trap catches can be highly variable throughout landscapes, so we investigated the potential impacts of environmental factors on adult *Ae*. *aegypti* capture rates during a “rear and release” program in California to better understand the relative contributions of true variability in population density across a landscape and trap context. We recorded male and female *Ae*. *aegypti* catches from BGS traps, with and without CO_2_, throughout control sites where no mosquitoes were released and in treatment sites where males infected with *Wolbachia* were released. BGS trap catches were positively influenced by higher proportions of shade or bushes in the front yard of the premises as well as the presence of potential larval habitats such as subterranean vaults. In contrast, an increase in residential habitat within a 100 m radius of trap locations negatively influenced BGS trap catches. For male *Ae*. *aegypti*, increased visual complexity of the trap location positively influenced capture rates, and the presence of yard drains negatively affected catch rates in control sites. Lastly, for BGS traps using CO_2_, higher catch rates were noted from traps placed greater than one meter from walls or fences for both male and female mosquitoes. These results have important implications for surveillance programs of *Ae*. *aegypti* throughout the Californian urban environment including adult monitoring during “rear and release” programs.

## Author summary

The efficacy of the Biogents Sentinel traps, the gold standard for catching *Aedes aegypti* mosquitoes, is impacted by various factors. We investigated environmental influences on BGS trap catches of *Ae*. *aegypti* during a “rear and release” program using control sites, not containing manipulated populations, and treatment sites where males infected with *Wolbachia* were released. We found BGS trap catches to be influenced by premises vegetative characteristics, potential larval habitats, trap placement factors and the amount of residential habitat surrounding trap positions. These effects varied depending on the mosquito sex, whether the trap used CO_2_ and whether the population was impacted by male releases. Our findings can inform a variety of programs requiring the surveillance of adult *Ae*. *aegypti*.

## Introduction

*Aedes aegypti*, the main vector of dengue, Zika, chikungunya and yellow fever viruses continues to increase its global distribution invading urban habitats outside of tropical climates, including many locations within the USA [1]. *Aedes aegypti* spread rapidly throughout North America being recorded in 220 counties in 28 states and the District of Columbia between 1995 and 2017 and was even responsible for a Zika virus outbreak in Florida in 2016 [2]. Within California, *Ae*. *aegypti* were introduced multiple times over the last few decades [3] with breeding populations being established in 2013 in Madera and Fresno in the central valley and San Mateo on the coast [4]. Based on typical trends [5], we could expect California to experience local outbreaks of diseases vectored by *Ae*. *aegypti* within the next decade. As *Ae*. *aegypti* have yet to reach their projected distributional limit within the USA [1, 5], this species is of great concern to many mosquito abatement districts.

With the relatively recent invasion of *Aedes* into California, traditional native mosquito control methods in this state have been heavily revised [6] to include treatments targeting these species. Recently, there has been significant global interest in the development of “rear and release” [7] programs to control mosquito populations [8–12]. These control methods involve mass-rearing mosquitoes in laboratories and releasing them throughout a landscape to either replace (using transinfections of symbiotic bacteria, such as *Wolbachia*; [12, 13]) or suppress (using the sterile insect technique or incompatible insect technique (IIT)) local populations [8, 9]. Verily Life Sciences (a Google affiliate) have been successfully performing an IIT “rear and release” program in Fresno County, California using male *Ae*. *aegypti* mosquitoes infected with *Wolbachia* which mate with local females and produce non-viable offspring due to cytoplasmic incompatibility [14]. By releasing 14.4 million males infected with *Wolbachia* across three neighbourhoods in 2018 Verily Life Sciences were able to demonstrate a suppression rate during the peak mosquito season of 95.5% [14].

Rear and release programs commonly deploy the Biogents Sentinel (Regensburg, Germany; hereinafter BGS) trap to survey mosquito populations and monitor success [8, 9, 12, 15]. The BGS trap is known as the gold standard for sampling *Aedes* mosquitoes and has been shown to outperform many other traps [16, 17]. This trap can be deployed unbaited, relying on its dark colouration to visually attract *Aedes* or with olfactory attractants such as BG-Lures and/or CO_2_ [18, 19]. It is common practice to use CO_2_ in combination with the BG-Lure in BGS traps as studies indicate that such methodology significantly increases *Ae*. *aegypti* catches [20, 21].

Despite the success of mosquito traps, including the BGS trap, *Ae*. *aegypti* collection rates in these traps can be highly variable [6]. Trap catches may be influenced by trap placement within premises and the attraction of mosquitoes to nearby oviposition and blood-feeding opportunities [6]. The distribution and dispersal of *Ae*. *aegypti* throughout the urban landscape may also be influenced by the availability of breeding habitats, barriers such as unshaded areas, busy roads or water bodies [22–24] which in turn could potentially impact collection rates. It is clear that identifying the causes of variation in BGS trap capture rates is vital to understanding and monitoring population trends across landscapes.

To date, few studies have evaluated the effects of environmental influences on BGS trap collection rates. An exception is recent work by Staunton, Yeeles (15), which evaluated the influences of premises condition and trap location factors during the Debug Innisfail “rear and release” program in northern Australia (https://research.csiro.au/mozzieproject/). Staunton, Yeeles (15) found that, in control sites (where male mosquitoes were not released), the condition of a house and whether or not it had screened windows influenced trap catch rates, as well as trap location factors such as the shelter above the trap, distance from outside sitting areas, and the visual complexity of the trap environment. In suburbs where *Wolbachia*-infected male mosquitoes were released, Staunton, Yeeles (15) detected influences in BGS trap catches, relative to control sites, from the amount of shade within a premises, as well as the house design and building materials.

While the findings of Staunton, Yeeles (15) are informative for the northern Australian urban environment, they may not directly apply to the Californian *Ae*. *aegypti* habitat. Unlike the urban environment of northern Australia, California displays cold wet winters and the summer is dry and hot, with low humidity—environmental factors once thought to inhibit the establishment of *Ae*. *aegypti* in California [6]. Since rainfall in the Central Valley of California is restricted to cold months of the year when temperatures are unsuitable for larval development, larval habitats are reliant on water from residential watering [25]. However, similar to northern Australia, larval habitats for *Ae*. *aegypti* in California may also consist of surface water-holding containers as well as subterranean sites [6, 26]. Adult mosquito harbourage in northern Australia is often associated with “Queenslander” styled homes which may be unscreened, in relatively poor condition and contain ground-level open access rooms [27, 28]. As such houses are uncommon in California, adult mosquito harbourage may instead be linked to favourable microclimates provided by vegetative features and residential watering, as suggested by Hayden, Uejio [29] for *Ae*. *aegypti* in Arizona.

We investigated the influences of environmental factors on male and female *Ae*. *aegypti* catches in BGS traps, with and without CO_2_, during Verily’s 2018 “rear and release” program in Fresno County, California. The environmental factors assessed relate to vegetation characteristics, potential larval habitats, trap placement characters, and the amount of residential environment surrounding the trap. This investigation not only gives insight into the ecology of this species, but also valuable information for designing surveillance programs in such urban habitats with or without male releases.

## Methods

### Male mass rearing and release

Infected *Ae*. *aegypti* were mass-reared in the Verily facility in South San-Francisco as described by [14]. Female mosquitoes were removed prior to release using Verily’s automated sex-sorting process. On average, 78,000 males were consistently released daily between 17 April– 17 October 2018 across all treatment areas from a specially modified van that tracked the GPS location and size of each release. See Crawford, Clarke [14] for further details regarding this program.

### Climate

Fresno County summers are generally characterised as hot with no rainfall and low humidity. Between July and October 2018, temperatures ranged from 56 to 108 F. The total rainfall during this period was 0 mm (NOAA weather station, Fresno Airport).

### Response data collection and selection

The premises and trap evaluation protocol involved visually inspecting 180 BGS traps throughout six communities in Fresno County (for map refer to Crawford, Clarke [14]) between 12 and 14 September 2018. To maintain consistency of assessment methodology, a single staff member was responsible for scoring predictors for all traps and only traps within domestic-styled housing locations were analysed. BGS traps assessed within control sites (n = 68) were located in locations labelled C1 (n = 25), C2 (n = 28) and C3 (n = 15) which were 66 ha, 66 ha and 46 ha in area, respectively. These control sites were geographically paired with treatment sites (n = 112) in T1 (n = 44), T2 (n = 33) and T3 (n = 35), which were 130 ha, 74 ha and 89 ha in area, respectively (for further details including a map see Crawford, Clarke [14]). Each site was subdivided into 7-acre grids, and BGS traps were randomly set within grid cells in order to maintain even coverage and consistent trap densities among sites [14]. As the program aim was to suppress *Ae*. *aegypti* populations, an operational decision was made to release more males in locations with higher housing densities, assessed in this study as residential habitat, as such locations were thought to contain larger populations of this mosquito. Additionally, trap locations were biased towards houses which contained vegetation in their front yards (a predominant characteristics of such neighbourhoods) and traps were often positioned behind vegetation to improve security from passers-by. The data analysed were selected from six months of trapping, which coincides with state-wide peaks in activity [6], when both male and female mosquitoes were most active between 1 May– 1 November 2018. At all times BGS traps were baited with BG-Lures (Biogents, Regensburg, Germany), but within each weekly cycle all BGS traps were run simultaneously for six nights without CO_2_ and then one night with CO_2_, (deployed using 2 pounds of dry ice per trap held in an ‘Insulated Dry Ice Bucket for EVS Trap’ purchased from Bioquip, California, USA). Therefore, the four datasets analysed were total male and female *Ae*. *aegypti* collections from BGS traps set with and without CO_2_.

### Environmental factor selection

Through consultation among mosquito experts, including local abatement staff, we selected and recorded a range of environmental and trap location factors considered relevant to this urban habitat (S1 Table). First, we recorded factors characterising the vegetative structure within a property. These included: the level of shade present in the yard (similar to that proposed by Tun-Lin, Kay [30] where scores 1–3 represented the proportion of yard covered in vegetative shade as <25%, 25–50% or >50%, respectively; S1 Fig), the proportion of yard that contained bushes as either being (<33%, 33–66% and >66% with scores 1–3 respectively; S2 Fig) and the number of neighbours surrounding the premises which contained high levels (>50%) of shade. Unlike Tun-Lin, Kay (30) we did not score house condition due to the consistently good condition of dwellings in the study sites [25].

Second, we recorded the within-premise factors characterising the traps’ locations. These included: whether the visibility of the trap from the road was clear, partially obscured or completely obscured (S3 Fig), the distance the trap was positioned from a wall or fence structure (<1 m, 1–2 m & >2 m; S4 Fig) and the visual complexity surrounding the trap from either competition with surrounding dark objects [18] and/or general visual obstruction (low, medium and high; S5 Fig). Third, we recorded various types of potential larval habitats (through exposure to water from irrigation) found throughout the premises. We noted the number of yard containers such as: buckets, tires, potted plant bases and tarpaulins within a premises as well as the presence of Pacific Gas and Electric (PG&E) subterranean vaults, yard drains and catch basins (S6 Fig). Finally, we investigated the influence of the amount of residential habitat within a 100 m radius of the trap location. These proportions were calculated by first buffering building footprints by 5 m, and then for each trap calculating the percentage of a 100 m radius circle around it that was covered by the buffered building area (S7 Fig).

### Premises condition and trap location data analysis

Models were run within the R statistical environment ver. 3.3.3 (R Core Team 2017) using a methodology previously reported by Staunton, Yeeles (15). We created separate models for male and female response data (total counts per trap), each including both the pooled treatment (n = 112) and control (n = 68) sites, using generalised linear models (GLM) in the *lme4* package [31]. Differences between continuous predictors were scaled (2*SD) and centred to avoid convergence issues [32]. Initial count-data models fit using Poisson distributions exhibited overdispersion, therefore final models were estimated using negative binomial distributions with log-link functions. We used the ‘*corvif*’ function in the *AED* package [33] to calculate variance-inflation factors and found that multicollinearity wasn’t likely to confound models. Subsequently, we analysed the effect of 11 parameters (shade, proportion of bushes in front yard, number of neighbours with high shade level in their premises, the visibility of the trap from the road, the distance of the trap from a wall or fence, the visual complexity of a trap, the number of yard containers, the presence of PG&E vaults, yard drains and catch basins and the amount of residential habitat within a 100 m radius of the trap) that might influence trap capture rates and their interactions with treatment (the releases of males). To account for trap fails we included an offset parameter in the model of the count of the total number of trap successes during the sampling period analysed. Finally, to account for the bias created by releasing more males in locations with higher housing densities we included a covariate of the total number of males released within 100 m radius of each BGS trap during the collection period as a fixed factor in our models. This factor was calculated for each release using a sensor on board the release vehicle that counts each individual mosquito as it is released and creates a record with GPS coordinates. In the rare instances where the sensor failed, the data was backfilled with a point placed at the triggered release location equal to the number of mosquitoes placed into the release device at the factory.

A single model would therefore take the basic form of, for example, Total female count per trap ~ (11 Parameters) * Treatment + Males released within 100 m of BGS trap locations + offset (trap successes). Using this approach, influences from parameters on response variables of interest from control sites could be modelled with interactions with male releases in treatment sites. By incorporating these interactions into the models we could examine how changes in environmental characteristics influenced catch rates per trap within Control sites, and whether they were relatively different in Treatment sites. We used backward selection to simplify models [34] by comparing AIC (Akaike’s Information Criterion) scores. Finally, we sequentially reintegrated removed terms back into the final model to determine their effect sizes and prevent bias associated with otherwise assigning effect sizes of zero [35, 36]. As the model used a negative bionomial distribution with a log-link function, Pseudo-R^2^ (McFadden) values [37] were calculated for final models using the *modEvA* package [38] to indicate model fit. The McFadden Pseudo-R2, calculated as 1 –(the maximum of the log likelihood function divided by the log likelihood of the null model), describes an excellent model fit being between 0.2 and 0.4 [39].

A linear regression was performed (in GraphPad Prism ver. 7.03) to confirm our suspected bias between the proportion of residential habitat within a 100 m radius of trap locations and the total number of males released per BGS trap assessed. For this analysis, data from both trap types (with and without CO_2_) were combined as the released males potentially lived longer than 24 hours and therefore males may have been caught by either trap type.

## Results

### Trap catches

In total, 604,262 *Ae*. *aegypti* were sampled over the six month period from the 180 BGS traps analysed in this study (Table 1).

**Table 1:** BGS trap collections of *Ae*. *aegypti* in Fresno County from 1 May—1 November 2018. Traps ran for a total of 27 weeks in this analysis and during that time were serviced either after one (traps with CO_2_) or six days (traps without CO_2_) per week. Male *Ae*. *aegypti* infected with *Wolbachia* were not released in control sites, but were released within treatment sites.

	Male			Female	
	with CO2	without CO2		with CO2	without CO2
**Total catch**					
Control	10,163	8,495		14,372	17,547
Treatment	206,707	342,519		2,011	2,448
**Mean total catch per trap (±S.E.)**					
Control	149.5 (21.8)	124.9 (16.4)		211.4 (19.7)	258.0 (21)
Treatment	1,845.6 (92)	3,058.2 (176.1)		18.0 (2.3)	21.9 (2.3)
**Mean catch per trap week (±S.E.)**					
Control	5.5 (0.8)	4.6 (0.6)		7.9 (0.7)	9.6 (0.8)
Treatment	68.4 (3.4)	113.3 (6.5)		0.7 (0.1)	0.8 (0.01)
**Mean catch per trap day (±S.E.)**					
Control	5.5 (0.8)	0.8 (0.1)		7.9 (0.7)	1.6 (0.1)
Treatment	68.4 (3.4)	18.9 (1.1)		0.7 (0.1)	0.13 (0.01)

### Bias associated with releasing more males in areas with greater residential habitat

There was a significantly positive relationship (R^2^ = 0.37, *F*_1,110_ = 64.1, *P* < 0.0001) between the proportion of residential habitat within a 100 m radius of trap locations and the total number of males released per BGS trap (S8 Fig).

### Influences of environmental factors on male *Ae*. *aegypti* catches in BGS traps with CO_2_

**Fig 1:**
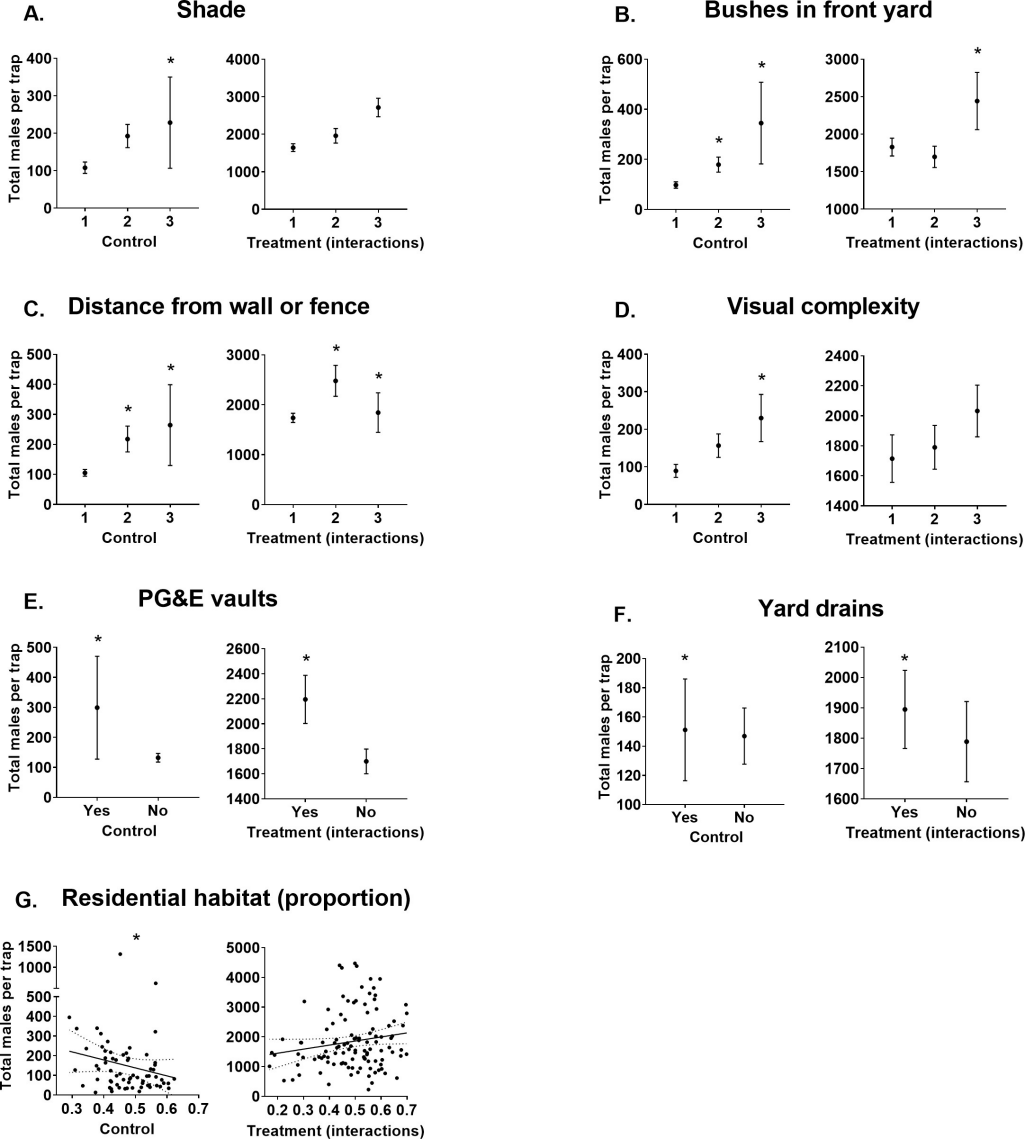
Total male *Ae*. *aegypti* catches per BGS trap baited with CO_2_ (ß ± S. E.) for each predictor with confidence intervals that don’t overlap zero. Predictors grouped as those from sites which didn’t have males released (Control) and those which interacted with males released (Treatment (interactions)). Stars indicate modelled predictor levels with confidence intervals that don’t overlap zero. Note that treatment interaction data may be biased by greater male releases occurring in areas with higher residential habitat densities which is accounted for in the model. A) Shade levels 1, 2 & 3 represent <25%, 25–50% & >50% coverage of yard by shade, respectively. B) Bushes in front yard levels 1, 2, & 3 represent <33%, 33–66% and >66%, respectively. C) Distance of the trap from a wall or fence levels 1, 2 & 3 represent <1 m, 1–2 m, & >2 m, respectively. D) Visual complexity levels 1, 2 & 3 represent clear, partial and high amounts of obstruction and/or visual competition from surrounding features, respectively. E) If PG&E vaults were present at the premises. F) Whether yard drains were present at the premises. G) Proportion of residential habitat (m^2^) within a 100 m radius of trap locations.

Releasing males positively influenced (Treatment: 1.92 ± 0.26 [ß ± S. E.] & Males released within 100 m radius: 1.04 ± 0.18; Table 2) male catches in BGS traps baited with CO_2_. Additionally, BGS traps located in premises with high (>50%) levels of shade caught more males (0.33 ± 0.13; Table 2; Fig 1A) than those located in premises containing low shade (<25%). Vegetation further influenced BGS catches with more males being sampled in traps at premises with medium (33–66%) or high (>66%) proportions of bushes in the front yard in control sites (0.32 ± 0.14 and 0.8 ± 0.22 respectively; Table 2; Fig 1B). However, the positive influence from a high proportion of bushes on BGS trap catches was not supported in treatment sites (-0.66 ± 0.27; Table 2; Fig 1B).

**Table 2:** Parameter estimates from the GLMs fitting male and female *Ae*. *aegypti* catches per BGS trap baited with CO_2_. Effects with ‘: Treatment’ refer to interactions between parameters and the treatment (male *Ae*. *aegypti* infected with *Wolbachia* release) effect. Pseudo-R^2^ (McFadden) values = 0.28 and 0.3 for males and female models, respectively.

Model treatment effects	Males		Females	
	Estimate	S. E.	Estimate	S. E.
Intercept	**1.65**	**0.19**	**1.69**	**0.10**
Treatment yes	**1.92**	**0.26**	**-2.46**	**0.11**
Shade 2	0.15	0.10	**0.33**	**0.12**
Shade 3	**0.33**	**0.13**	**0.53**	**0.15**
Bushes in front yard medium	**0.32**	**0.14**	0.09†	0.11
Bushes in front yard high	**0.80**	**0.22**	-0.05†	0.19
Neighbours with high shade	-0.02†	0.09	-0.06†	0.11
Trap visibility from road partially obscured	-0.05†	0.15	0.08†	0.16
Trap visibility from road obscured	-0.08†	0.14	0.04†	0.17
Trap distance from wall or fence 1–2 m	**0.65**	**0.16**	**0.58**	**0.14**
Trap distance from wall or fence >2 m	**0.49**	**0.18**	**0.64**	**0.17**
Visual complexity 2	0.14	0.15	0.06†	0.13
Visual complexity 3	**0.63**	**0.21**	0.22†	0.14
PG&E vault yes	**0.47**	**0.20**	**0.36**	**0.13**
Yard drains yes	**-0.27**	**0.13**	-0.13†	0.11
Catch basin yes	0.12†	0.14	-0.17†	0.20
Yard containers 2	0.03†	0.20	0.12†	0.27
Yard containers 3	0.31†	0.17	0.25†	0.23
Residential habitat within 100 m radius	**-0.37**	**0.12**	**-0.57**	**0.13**
Males released within 100 m radius	**1.04**	**0.18**	0.28†	0.24
Shade 2: Treatment yes	-0.05†	0.20	-0.17†	0.25
Shade 3: Treatment yes	0.04†	0.26	-0.03†	0.30
Bushes in front yard medium: Treatment yes	-0.29	0.18	-0.28†	0.22
Bushes in front yard high: Treatment yes	**-0.66**	**0.27**	-0.48†	0.35
Neighbours with high shade: Treatment yes	-0.04†	0.22	-0.10†	0.23
Trap visibility from road partially obscured: Treatment yes	-0.19†	0.30	0.10†	0.32
Trap visibility from road obscured: Treatment yes	-0.45†	0.28	0.05†	0.33
Trap distance from wall or fence 1–2 m: Treatment yes	**-0.46**	**0.21**	0.09†	0.28
Trap distance from wall or fence >2 m: Treatment yes	**-0.55**	**0.25**	0.51†	0.33
Visual complexity 2: Treatment yes	0.07	0.20	-0.03†	0.25
Visual complexity 3: Treatment yes	-0.39	0.25	0.03†	0.32
PG&E vault yes: Treatment yes	**-0.52**	**0.23**	-0.15†	0.30
Yard drains yes: Treatment yes	**0.50**	**0.16**	0.07†	0.21
Catch basin yes: Treatment yes	0.24†	0.32	0.30†	0.41
Yard containers 2: Treatment yes	0.10†	0.42	-0.68†	0.59
Yard containers 3: Treatment yes	-0.39†	0.35		-0.31†	0.44
Residential habitat within 100 m radius: Treatment yes	0.07†	0.27		-0.10†	0.32
AICc null model	2,908			1,941	
AICc final model	2,589			1,656	

Notes: † = term removed during backward selection and reintegrated into final model to determine effect size. Parameter estimates with 95% confidence intervals that do not overlap zero are in boldface type.

BGS traps with CO_2_ that were positioned within premises either 1>2 m or >2 m from a wall or fence displayed higher catch rates of male *Ae*. *aegypti* in control sites (0.65 ± 0.16 and 0.49 ± 0.18 respectively; Table 2; Fig 1C). Both these relationships were not supported with treatment sites (-0.46 ± 0.21 and -0.55 ± 0.25, respectively; Table 2; Fig 1C). Additionally, BGS traps located in highly visually complex areas caught more males than those set in locations characterised by low visual complexity (0.63 ± 0.21; Table 2; Fig 1D).

PG&E vault presence positively influenced male catches in BGS traps with CO_2_ (0.47 ± 0.20; Table 2; Fig 1E), although this relationship was not supported in treatment sites (-0.52 ± 0.23; Table 2; Fig 1E). Yard drain presence negatively influenced BGS trap (with CO_2_) catches of male *Ae*. *aegypti* in controls sites (-0.27 ± 0.13; Table 2; Fig 1F), but again this relationship was not supported in treatment sites (0.50 ± 0.16; Table 2; Fig 1F). Finally, the amount of residential habitat, within a 100 m radius of each trap, negatively influenced (-0.37 ± 0.12) male catches in control sites (Table 2; Fig 1F).

### Influences of environmental factors on female *Ae*. *aegypti* catches in BGS traps with CO_2_

**Fig 2:**
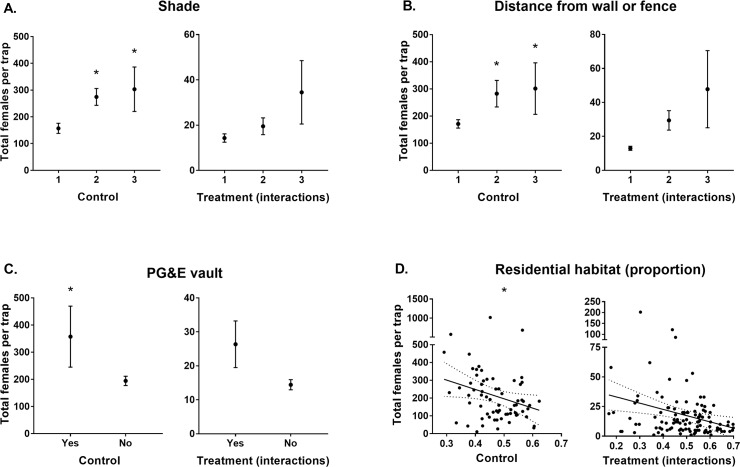
Total female *Ae*. *aegypti* catches per BGS trap baited with CO_2_ (ß ± S. E.) for each predictor with confidence intervals that don’t overlap zero. Predictors grouped as those from sites which didn’t have males released (Control) and those which interacted with males released (Treatment (interactions)). Stars indicate modelled predictor levels with confidence intervals that don’t overlap zero. Note that treatment interaction data may be biased by greater male releases occurring in areas with higher residential habitat densities which is accounted for in the model. A) Shade levels 1, 2 & 3 represent <25%, 25–50% & >50% coverage of yard by shade, respectively. B) Distance of trap from a wall or fence levels 1, 2 & 3 represent <1 m, 1–2 m, & >2 m, respectively. C) If PG&E vaults were present at the premises. D) Proportion of residential habitat (m^2^) within a 100 m radius of trap locations.

Releasing *Wolbachia*-infected males negatively influenced (-2.46 ± 0.11; Table 2) female catches in BGS traps baited with CO_2_. BGS traps located in premises with medium (25–50%) or high (>50%) levels of shade caught more females (0.33 ± 0.12 and 0.53 ± 0.15 respectively; Table 2; Fig 2A) than those located in premises with low shade (<25%).

BGS traps distanced either 1–2 m or >2 m from a wall or fence caught more female *Ae*. *aegypti* than those within 1 m of such structures (0.58 ± 0.14 and 0.64 ± 0.17 respectively; Table 2; Fig 2B). Again, the presence of PG&E vaults positively influenced BGS trap catches (0.36 ± 0.13; Table 2; Fig 2C). Lastly, the amount of residential habitat within a 100 m radius of each trap negatively influenced (-0.57 ± 0.13) female catches (Table 2; Fig 2D).

### Influences of environmental factors on male *Ae*. *aegypti* catches in BGS traps without CO_2_

**Fig 3:**
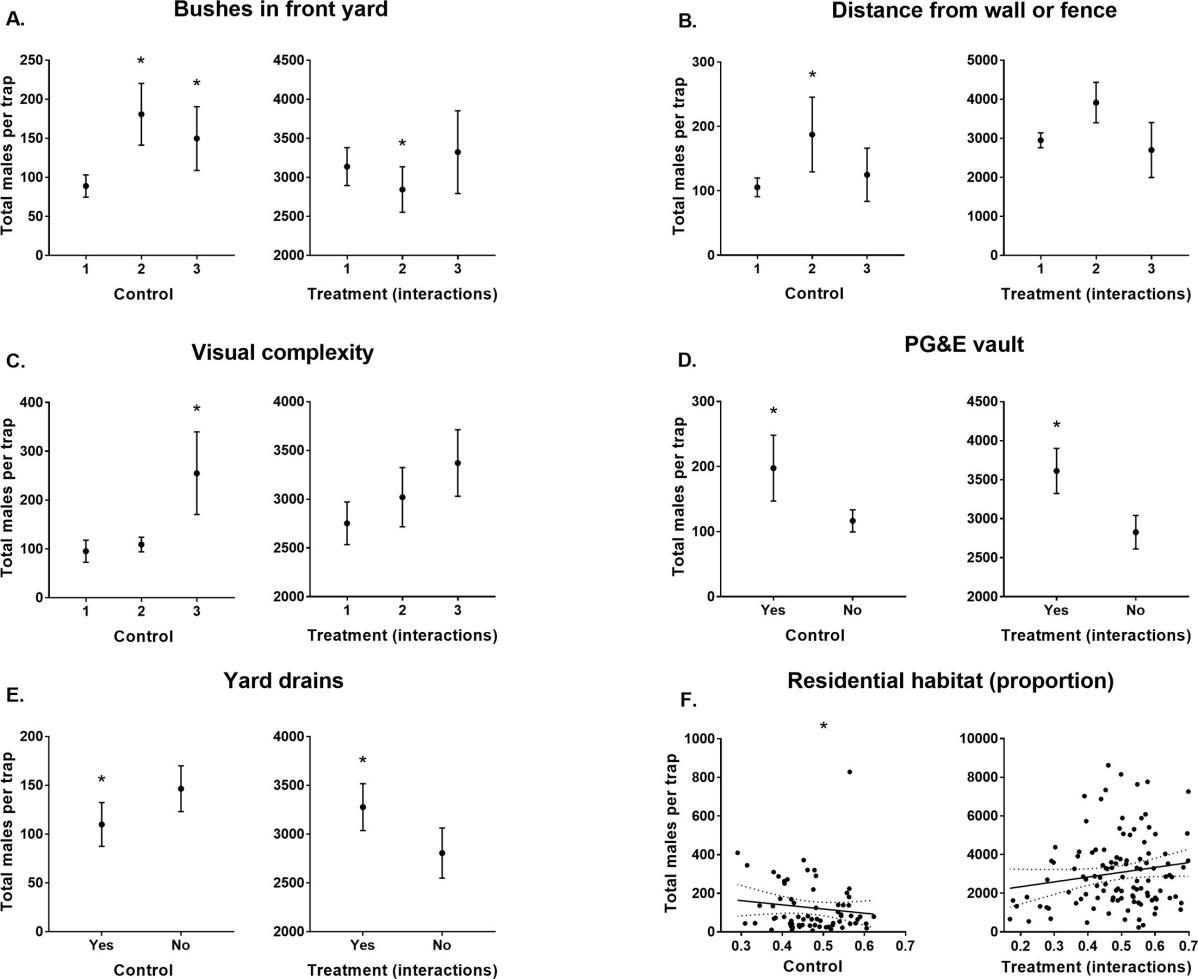
Total male *Ae*. *aegypti* catches per BGS trap without CO_2_ (ß ± S. E.) for each predictor with confidence intervals that don’t overlap zero. Predictors grouped as those from sites which didn’t have males released (Control) and those which interacted with males released (Treatment (interactions)). Stars indicate modelled predictor levels with confidence intervals that don’t overlap zero. Note that treatment interaction data may be biased by greater male releases occurring in areas with higher residential habitat densities which is accounted for in the model. A) Bushes in front yard levels 1, 2, & 3 represent <33%, 33–66% and >66%, respectively. B) Distance of the traps from a wall or fence levels 1, 2 & 3 represent <1 m, 1–2 m, & >2 m, respectively. C) Visual complexity levels 1, 2 & 3 represent clear, partial and high amounts of obstruction and/or visual competition from surrounding features, respectively. D) If PG&E vaults were present at the premises. E) Yard drains present or absent at premises. F) Proportion of residential habitat (m^2^) within a 100 m radius of trap locations.

Releasing males positively influenced (Treatment: 2.29 ± 0.33 & Males released within 100 m radius: 1 ± 0.24; Table 3) the numbers of males caught in BGS traps that were not baited with CO_2_. BGS traps located at premises with medium (33–66%) or high (>66%) proportions of bushes in the front yard in control sites caught more males than those with low (<30%) proportions of bushes (0.56 ± 0.18 and 0.77 ± 0.27 respectively; Table 3; Fig 1A). However, the positive influence from placing traps in locations with medium proportions of bushes (33–66%) was not supported in treatment sites (-0.59 ± 0.22; Table 3; Fig 3A).

**Table 3:** Parameter estimates from the GLMs fitting male and female *Ae*. *aegypti* catches per BGS trap without CO_2_. Effects with ‘: Treatment’ refer to interactions between parameters and the treatment (*Wolbachia*-infected male *Ae*. *aegypti* releases) effect. Pseudo-R^2^ (McFadden) values = 0.22 and 0.25 for males and female models respectively.

Model treatment effects	Males		Females	
	Estimate	S. E.	Estimate	S. E.
Intercept	**1.80**	**0.23**	**2.06**	**0.14**
Treatment yes	**2.29**	**0.33**	**-2.32**	**0.15**
Shade 2	0.08†	0.12	**0.35**	**0.14**
Shade 3	0.11†	0.16	**0.42**	**0.18**
Bushes in front yard medium	**0.56**	**0.18**	0.27	0.19
Bushes in front yard high	**0.77**	**0.27**	-0.13	0.30
Neighbours with high shade	-0.02†	0.12	-0.16†	0.12
Trap visibility from road partially obscured	0.08†	0.19	-0.08†	0.18
Trap visibility from road obscured	-0.04†	0.18	-0.06†	0.18
Trap distance from wall or fence 1–2 m	**0.32**	**0.14**	0.23	0.15
Trap distance from wall or fence >2 m	-0.05	0.16	0.35	0.18
Visual complexity 2	-0.12	0.20	-0.06†	0.14
Visual complexity 3	**0.66**	**0.27**	0.13†	0.16
PG&E vault yes	**0.61**	**0.26**	**0.48**	**0.14**
Yard drains yes	**-0.33**	**0.16**	-0.18	0.11
Catch basin yes	0.31	0.19	-0.23†	0.21
Yard containers 2	-0.18†	0.26	0.12†	0.29
Yard containers 3	0.32†	0.22	0.22†	0.25
Residential habitat within 100 m radius	**-0.33**	**0.16**	**-0.55**	**0.15**
Males released within 100 m radius	**1.00**	**0.24**	0.02†	0.26
Shade 2: Treatment yes	-0.13†	0.25	-0.13†	0.28
Shade 3: Treatment yes	0.43†	0.33	0.29†	0.36
Bushes in front yard medium: Treatment yes	**-0.59**	**0.22**	-0.44	0.24
Bushes in front yard high: Treatment yes	-0.67	0.35	**-0.75**	**0.38**
Neighbours with high shade: Treatment yes	0.22†	0.27	0.09†	0.27
Trap visibility from road partially obscured: Treatment yes	-0.23†	0.38	0.29†	0.36
Trap visibility from road obscured: Treatment yes	-0.61†	0.36	-0.03†	0.36
Trap distance from wall or fence 1–2 m: Treatment yes	-0.24†	0.27	-0.15†	0.30
Trap distance from wall or fence >2 m: Treatment yes	-0.50†	0.33	0.63†	0.36
Visual complexity 2: Treatment yes	0.35	0.26	-0.15†	0.28
Visual complexity 3: Treatment yes	-0.35	0.32	0.39†	0.35
PG&E vault yes: Treatment yes	**-0.60**	**0.30**	0.13†	0.33
Yard drains yes: Treatment yes	**0.57**	**0.20**	0.26†	0.23
Catch basin yes: Treatment yes	-0.11†	0.41	-0.24†	0.45
Yard containers 2: Treatment yes	0.65†	0.55	-0.21†	0.63
Yard containers 3: Treatment yes	-0.09†	0.45		-0.07†	0.49
Residential habitat within 100 m radius: Treatment yes	0.11†	0.34		-0.40†	0.34
AICc null model	3,046			2,014		
AICc final model	2,751			1,762		

Notes: † = term removed during backward selection and reintegrated into final model to determine effect size. Parameter estimates with 95% confidence intervals that do not overlap zero are in boldface type.

BGS traps positioned at 1–2 m from a wall or fence displayed higher catch rates of male *Ae*. *aegypti* than those within 1 m of such structures (0.32 ± 0.14; Table 3; Fig 3B). Furthermore, BGS traps located in highly visually complex areas caught more males than those in locations characterised by low visual complexity (0.66 ± 0.27; Table 3; Fig 3C).

The presence of PG&E vaults positively influenced male catches in BGS traps without CO_2_ (0.61 ± 0.26; Table 3; Fig 3D). Whereas, the presence of yard drains within premises negatively influenced male catch rates (-0.33 ± 0.16; Table 3; Fig 3E). Both relationships, regarding these potential larval habitat types, were not supported in treatment sites (-0.6 ± 0.3; Table 3; Fig 3D and 0.57 ± 0.2; Table 3; Fig 3E for PG&E vaults and yard drains, respectively). Finally, the amount of residential habitat, within a 100 m radius of each trap, negatively influenced (-0.33 ± 0.16) male catches (Table 3; Fig 3F).

### Influences of environmental factors on female *Ae*. *aegypti* catches in BGS traps without CO_2_

**Fig 4:**
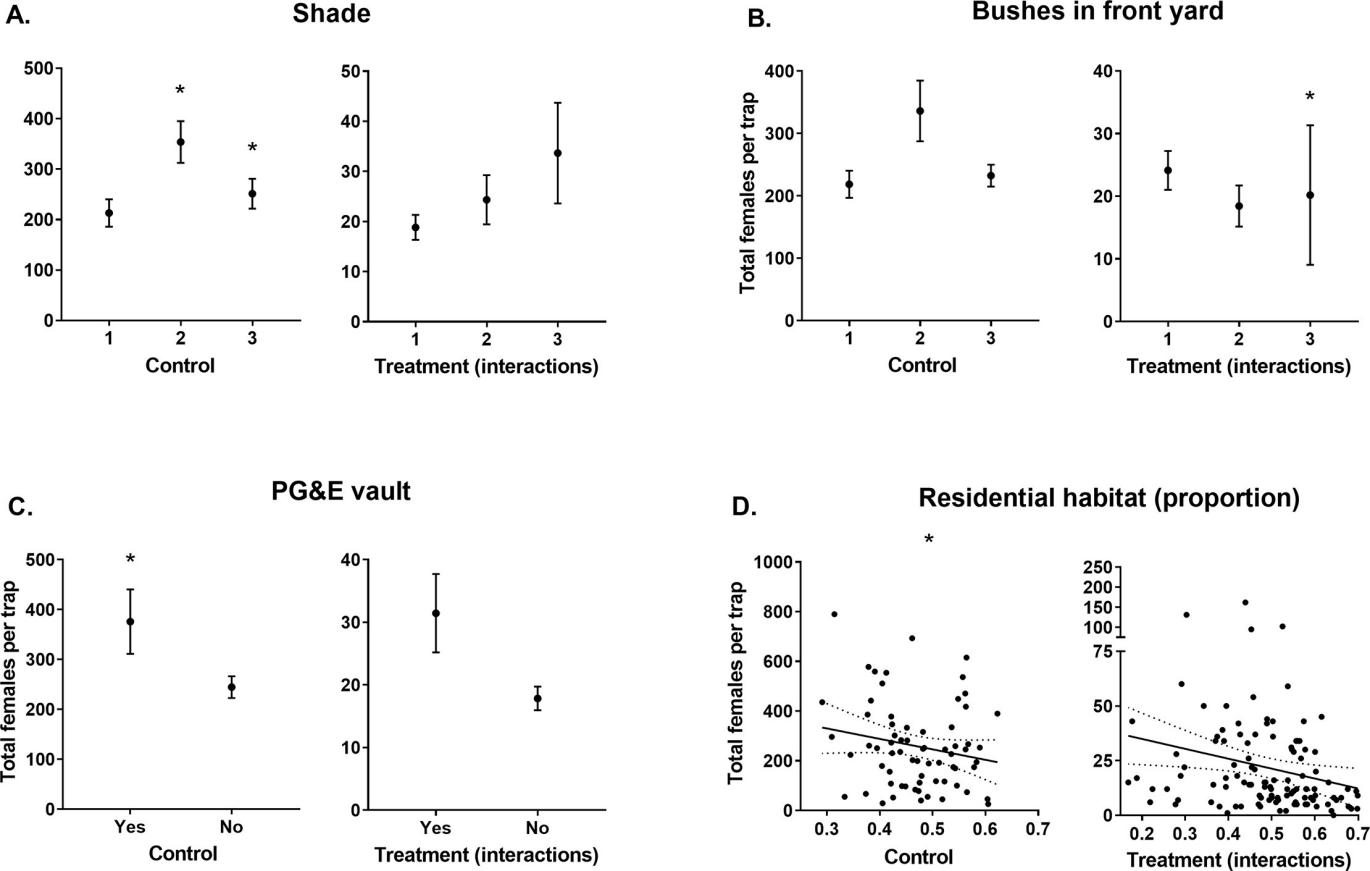
Total female *Ae*. *aegypti* catches per BGS trap without CO_2_ (ß ± S. E.) for each predictor with confidence intervals that don’t overlap zero. Predictors grouped as those from sites which didn’t have males released (Control) and those which interacted with released males (Treatment (interactions)). Stars indicate modelled predictor levels with confidence intervals that don’t overlap zero. Note that treatment interaction data may be biased by greater male releases occurring in areas with higher residential habitat densities which is accounted for in the model. A) Shade levels 1, 2 & 3 represent <25%, 25–50% & >50% coverage of yard by shade, respectively. B) Bushes in front yard levels 1, 2, & 3 represent <33, 33–66% and >66%, respectively. C) If PG&E vaults were present at the premises. D) Proportion of residential habitat (m^2^) within a 100 m radius of the trap location.

Releasing males negatively influenced (-2.32 ± 0.15; Table 3) female catches in BGS traps not baited with CO_2_. BGS traps located in premises with medium (25–50%) or high (>50%) levels of shade caught more females (0.35 ± 0.14 and 0.42 ± 0.18, respectively; Table 3; Fig 4A) than those in premises characterised by low shade (<25%).

Premises containing a high proportion (>66%) of bushes in the front yard in treatment sites negatively influenced female *Ae*. *aegypti* capture rates (from BGS traps without CO2), relative to such locations in control sites (-0.75 ± 0.38; Table 3; Fig 4B). The presence of PG&E vaults again positively influenced BGS trap catches of female *Ae*. *aegypti* (0.48 ± 0.14; Table 3; Fig 4C). Finally, the amount of residential habitat within a 100 m radius of each trap negatively influenced (-0.55 ± 0.15) female catches in BGS traps without CO_2_ (Table 3; Fig 4D).

## Discussion

Key findings from this study for consideration by staff setting BGS traps for *Ae*. *aegypti* surveillance in similar urban habitats include: 1) BGS traps placed within premisses with high proportions of shade, or medium to high proportions of bushes, caught more *Ae*. *aegypti* than those placed in locations where such features were scarce, 2) BGS traps deployed with CO_2_ displayed higher catch rates of *Ae*. *aegypti* when distanced >1 m from structures such as walls or fences, 3) BGS traps placed in locations characterised by high visual complexity, generally due to being placed behind bushes, demonstrated higher catch rates of male *Ae*. *aegypti* than those placed out in the open, 4) BGS traps placed within premisses with subterranean vaults, such as PG&E vaults, displayed higher catch rates of *Ae*. *aegypti* than those placed in premisses without such features and 5) BGS traps placed within locations surrounded by higher proportions of residential habitat displayed reduced catch rates of *Ae*. *aegypti*. Specific influences of the environmental factors mentioned above on BGS trap catch rates varied according to mosquito sex, if traps were deployed with or without CO_2_ and whether the population surveyed was impacted by male releases and are discussed in detail within the sections below.

### Influence from vegetation

Higher proportions of shade within premises positively influenced BGS trap catches of male *Ae*. *aegypti* when CO_2_ was used and females with or without the use of CO_2_. This association between shade and collection rates of *Ae*. *aegypti* has been previously recorded using ovitraps in Australia [24, 40] and in Arizona [29]. Staunton, Yeeles [15] found a positive association between high shade levels and both male and female *Ae*. *aegypti* catch rates in treatment sites for a similar rear and release program, although unlike this current work the relationship they detected wasn’t supported in the control sites. Well shaded areas have been suggested to provide favourable microclimates for resting *Ae*. *aegypti*, and may act as dispersal corridors and provide sustenance via floral nectaries to adults and decomposing organic matter for larvae [24, 29].

Our current study also found that medium or high proportions of bushes in the front yard of premises also positively influenced male *Ae*. *aegypti* catches in control sites. These relationships were less evident with BGS trap catches in treatment sites. Additionally, female catches using BGS traps without CO_2_ were negatively influenced by a high proportion of front yard bushes in the treatment sites, relative to the control sites. Like heavily shaded locations, bushy yards may provide suitable resting habitats for males which subsequently increase the catch rates of BGS traps located in such areas. However, when males are released from vans, a higher proportion of bushes may interfere with the movement of males into the BGS trap and thereby reduce catch rates relative to locations with comparatively fewer bushes. Additionally, BGS traps were commonly placed within or behind bushes in front yards. Therefore, a yard with fewer bushes may attract the males to the specific area containing this vegetation and subsequently, the BGS trap. It is unclear why comparatively fewer females were caught in treatment locations containing a greater proportion of bushes when using a BGS trap without CO_2_. Perhaps this was due to more effective suppression in such locations, but further investigations are certainly warranted to better clarify the influence of this environmental factor on *Ae*. *aegypti* catches.

### Distance from wall or fence

BGS traps with CO_2_ were generally more effective when placed further than 1 m from a wall or fence. The addition of CO_2_ to the BGS trap provides an olfactory attractant which increases the catch rates of BGS traps [21]. As the CO_2_ deployed with BGS traps was a gas likey carried by wind it is logical that placing the trap close to a barrier may have reduced the gas’s ability to permeate throughout a region and therefore decreased the trap’s attractive quality. The additional findings that trap location, regarding distance to a wall or fence, didn’t strongly influence catch rates when CO_2_ was not used, provide additional support for this hypothesis.

### Visual complexity

Placing the BGS traps in locations characterised by high levels of visual complexity positively influenced male catch rates. This finding contradicts previous work performed in northern Australia that suggests that higher visual complexity reduces the effectiveness of the BGS traps [15, 18]. While Staunton, Yeeles (15) suggested that this negative influence may derive from competing dark objects and obscured visual cues of the trap, the habitat in which the traps were placed in California is different to that of northern Australia. Unlike northern Australian premises, which may contain many manufactured objects in their yards [30], the premises in Fresno displayed front yards that generally contained manicured gardens. As stated above, traps were generally placed behind vegetation in the yard to reduce visibility to passers-by. Subsequently, the traps which were located within highly visually complex environments were generally also placed within bushy gardens. Although no collinearity between these factors was detected, traps which were hard to see may still have benefitted from being placed close to bushes which are likely to be attractive microhabitats.

### Potential larval habitats

Consistently, for both sexes and trap types, the presence of PG&E vaults positively influenced *Ae*. *aegypti* catch rates in control sites, potentially due to being productive larval habitats or effective harbourage features. The hot dry Californian summer climate was traditionally considered to inhibit *Ae*. *aegypti* establishment within this state [6]. However, within Los Angeles and Merida Mexico, *Ae*. *aegypti* were associated with storm sewers [6, 41] and research in northern Australia also emphasised the importance of subterranean containers, particularly telecommunication pits comparable to the PG&E vaults, as larval habitats [26]. In Australia, these underground larval habitats were increasingly used by *Ae*. *aegypti* during the dry winter when surface containers were dry [26]. Additionally, *Ae*. *aegypti* established within a highly xeric Indian town were noted to utilise subterranean cement water containers known as *tankas* [42], again indicating the potential importance of such features to this species’ survival.

Within treatment sites, the negative influence of PG&E vault presence on male catch rates, relative to control sites, may reflect the comprehensive release of males throughout neighbourhoods and even indicate effective suppression of such habitats. Clearly, further work is required to identify the extent to which these features are facilitating the establishment of *Ae*. *aegypti* within California and are able to be suppressed by male releases.

Unlike PG&E vaults, the presence of yard drains negatively influenced male *Ae*. *aegypti* catch rates in control sites. This finding was unexpected as, while not a direct focus of this study, our casual inspections of yard drains found them to generally contain water. Additionally, we noted that many wet yard drains contained yard clippings or other organic material and while we collected some *Ae*. *aegypti* larvae, in the few samples we collected, we predominantly found immature *Culex*. Although *Ae*. *aegypti* oviposition is known to increase in water containing an organic infusion [43], *Culex* are known to prefer to oviposit in water with a high organic content such as that from older infusions [44]. Therefore, perhaps yard drains are generally more suitable to sustaining *Culex* populations. As our observations regarding yard drains as larval habitats were relatively anecdotal, our BGS trapping results indicate that more formal studies into this larval habitat are warranted.

As with PG&E vaults, the influence of yard drain presence on male catch rates noted in control sites was not supported in treatment sites. Again, this difference in relationships between sites may relate to the comprehensive male releases throughout the treatment neighbourhoods. Female *Ae*. *aegypti* are thought to be attracted to the odours emanating from potential larval habitats [24] so it is possible that the males released from vans may also be attracted to such areas.

### Residential habitat with 100 m radius of trap

The finding that greater proportions of residential area negatively influenced *Ae*. *aegypti* catch rates in BGS traps is counter-intuitive to previous research demonstrating that these mosquitoes prefer densely occupied areas [45, 46]. Perhaps, the good condition of screened houses throughout these areas force these mosquitoes to display relatively exophilic behaviours. Therefore, trap catches may be positively influenced by alternate landscape features such as large yards, parks, car garages, roads and other clearings and buildings. Large yards and parks may contain additional features such as bushes or trees which create attractive microhabitats for *Ae*. *aegypti*. Additionally, clearings and busy roads are suggested to be barriers to dispersal for *Ae*. *aegypti* [22, 24] and as such may direct the movement of these mosquitoes towards locations containing the traps. These findings reinforce how environmental factors may influence *Ae*. *aegypti* catch rates at larger landscape scales and not just at fine resolutions within premisses.

It is worth restating that all traps and associated environmental factors were assessed within the front yards of premises. Unlike northern Australian communities, Californian residents do not support staff access to their backyards. While back yard features, which may influence BGS trap efficacy, were not assessed, the results presented in this study remain relevant and informative to public health staff who will generally set mosquito traps within front yards in this urban environment. Additionally, the authors acknowledge that fine-scale weather factors such as temperature and humidity at trap locations may also influence trap catch rates, but were not included in these analyses. Determining the influences of such factors were outside the scope of this study which aimed to provide information regarding environmental characteristics that were likely correlated, such as proportion of shade, but more easily applied by staff when selecting trap locations.

## Conclusions

The environmental factors that influenced BGS trap catches were generally consistent across mosquito sex and trap (with or without CO_2_) and site (control or treatment) type. Surveillance programs of *Ae*. *aegypti* within Fresno, should consider that BGS trap catches were positively influenced by the amount of vegetation within a premises and the presence of potential larval habitats or harbourage features such as PG&E vaults. Additionally, catch rates from BGS traps using CO_2_ were positively influenced when the traps were positioned >1 m away from walls or fences and male mosquito catches were strongly influenced by the proportion of bushes within premises, unlike females. Lastly, BGS trap catches were consistently negatively influenced by greater proportions of residential areas within 100 m of trap locations. Although it remains difficult to disentangle whether these environmental features are better indicators of local *Ae*. *aegypti* breeding and harbourage or better trap performance, such results indicate that male releases could be conditional, dependent on residential type and environmental characteristics such as vegetation structure and potential larval habitats.

These findings also suggest that environmental factors influence BGS trap catches of *Ae*. *aegypti* in California differently to tropical locations such as northern Australia and emphasise the highly adaptable nature of this species. Surveillance programs therefore need to strongly consider the fundamental requirements for each life stage of *Ae*. *aegypti* and how the environment they are surveying within may facilitate the survival of this species and consequently influence trap success.

## Supporting information

S1 Table Number of sites for each categorical predictor measured in control and treatment areas. (DOCX)

S1 Fig Different amounts of shade A. <25%, B. 25–50% C. >50%.
(TIF)

S2 Fig Different amounts of bushes in the front yard A. <33%, B. 33–66% and C. >66%.
(TIF)

S3 Fig BGS trap visibility from the road A. clear, B. partially obscured and C. obscured.
(TIF)

S4 Fig BGS trap proximity to wall or fence A. <1 m, B. 1–2 m and C. >2 m.
(TIF)

S5 Fig Visual competition to the BGS trap A. low, B. medium and C. high.
(TIF)

S6 Fig Potential larval habitats A. PG&E vault, B. yard drain, C. catch basin and D. yard container.
(TIF)

S7 Fig **Residential habitat footprints (yellow areas) within 100 m radius of BGS traps (blue points) in T1, Fresno.** Map created in QGIS version 3.4 with base layer sourced from The United States Geological Services (USGS).
(TIF)

S8 Fig Total males released per BGS trap for collections with and without CO_2_ combined (R^2^ = 0.37, *F*_1,110_ = 64.1, *P* < 0.0001). (TIF)
