# Supplementary material for: Environmental influences on Aedes aegypti catches in Biogents Sentinel traps during a Californian “rear and release” program: Implications for designing surveillance programs
Source: PLoS Negl Trop Dis. 2020 Jun 12;14(6):e0008367. doi: 10.1371/journal.pntd.0008367 (PMC7314095; doi:10.1371/journal.pntd.0008367)
Supplement: S1 Table — (DOCX) [file pntd.0008367.s001.docx]

**Table S1.** Number of sites for each categorical predictor measured in control and treatment areas.

| Predictor | number of sites | |
| --- | --- | --- |
|  | control | treatment |
| Treatment no | 68 | - |
| Treatment yes | - | 112 |
| Shade 1 | 39 | 74 |
| Shade 2 | 19 | 24 |
| Shade 3 | 10 | 14 |
| Bushes in front yard low | 39 | 64 |
| Bushes in front yard medium | 22 | 37 |
| Bushes in front yard high | 7 | 11 |
| Trap visibility from road obscured | 30 | 64 |
| Trap visibility from road partially obscured | 29 | 34 |
| Trap visibility from road clear | 9 | 14 |
| Trap distance from wall >1 m | 45 | 88 |
| Trap distance from wall 1-2 m | 14 | 15 |
| Trap distance from wall >2 m | 9 | 9 |
| Visual complexity 1 | 17 | 30 |
| Visual complexity 2 | 42 | 47 |
| Visual complexity 3 | 9 | 35 |
| PG&E vault yes | 7 | 33 |
| PG&E vault no | 61 | 79 |
| Yard drains yes | 40 | 60 |
| Yard drains no | 28 | 52 |
| Catch basin yes | 4 | 9 |
| Catch basin no | 64 | 103 |
| Yard containers 1 | 59 | 104 |
| Yard containers 2 | 5 | 2 |
| Yard containers 3 | 4 | 6 |
