# Supplementary figures and images for: Environmental influences on Aedes aegypti catches in Biogents Sentinel traps during a Californian “rear and release” program: Implications for designing surveillance programs
Source: PLoS Negl Trop Dis. 2020 Jun 12;14(6):e0008367. doi: 10.1371/journal.pntd.0008367 (PMC7314095; doi:10.1371/journal.pntd.0008367)

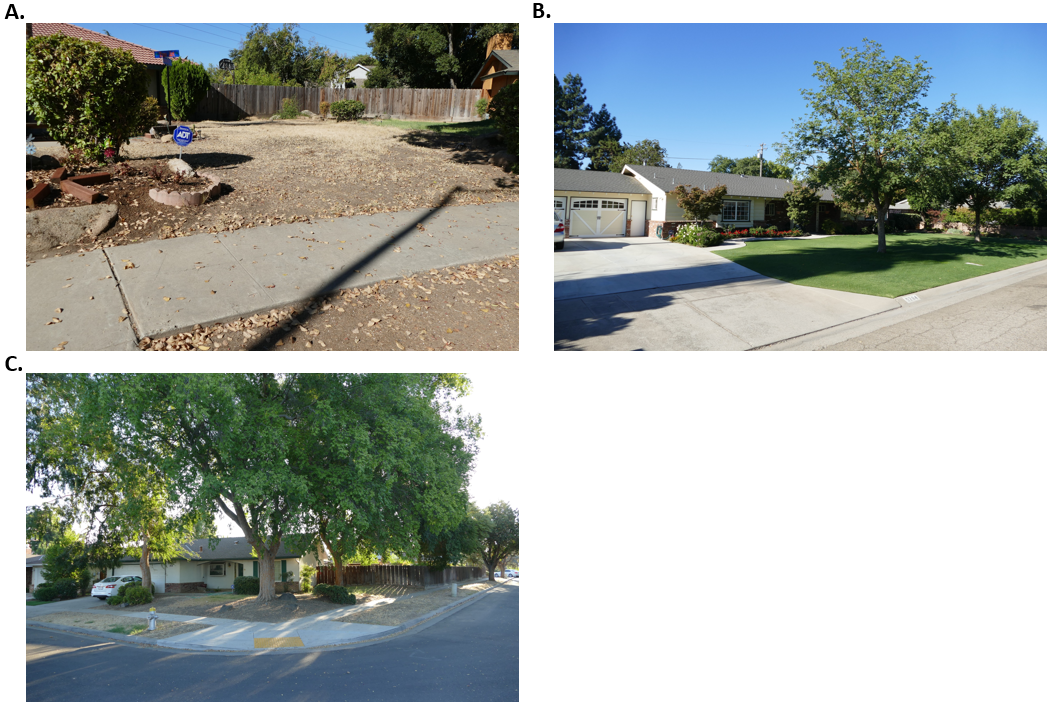

Supplement: S1 Fig — Different amounts of shade A. <25%, B. 25–50% C. >50%. (TIF) [file pntd.0008367.s002.tif]

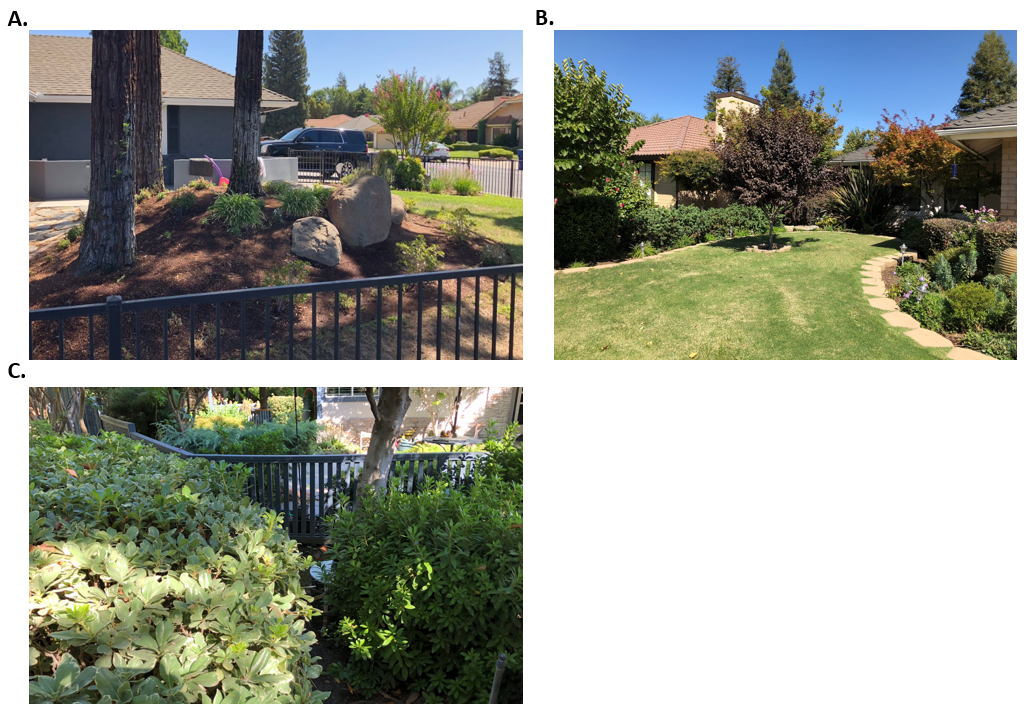

Supplement: S2 Fig — Different amounts of bushes in the front yard A. <33%, B. 33–66% and C. >66%. (TIF) [file pntd.0008367.s003.tif]

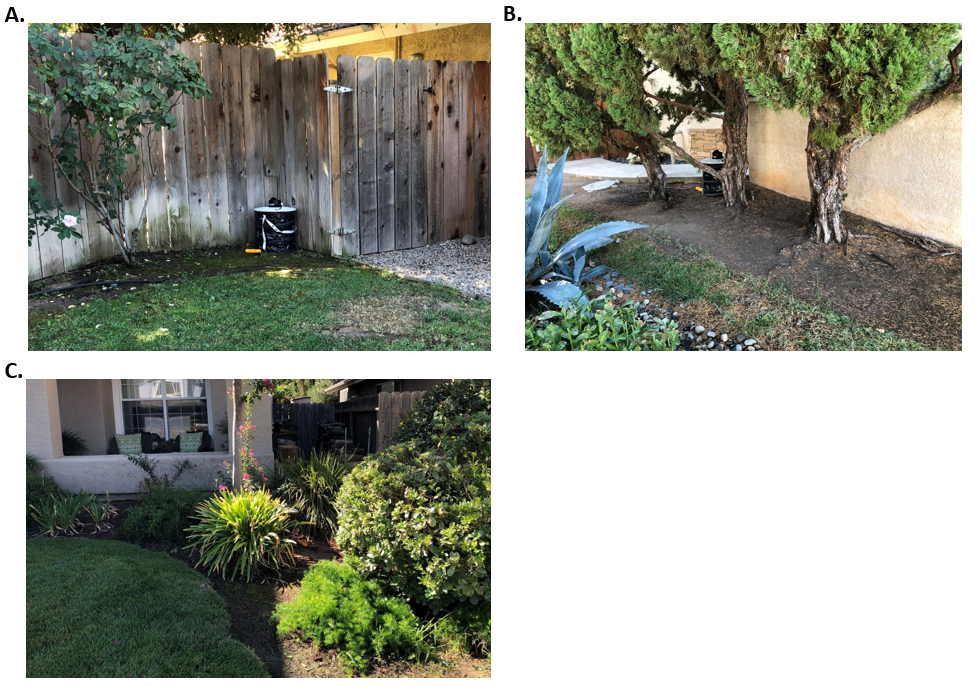

Supplement: S3 Fig — BGS trap visibility from the road A. clear, B. partially obscured and C. obscured. (TIF) [file pntd.0008367.s004.tif]

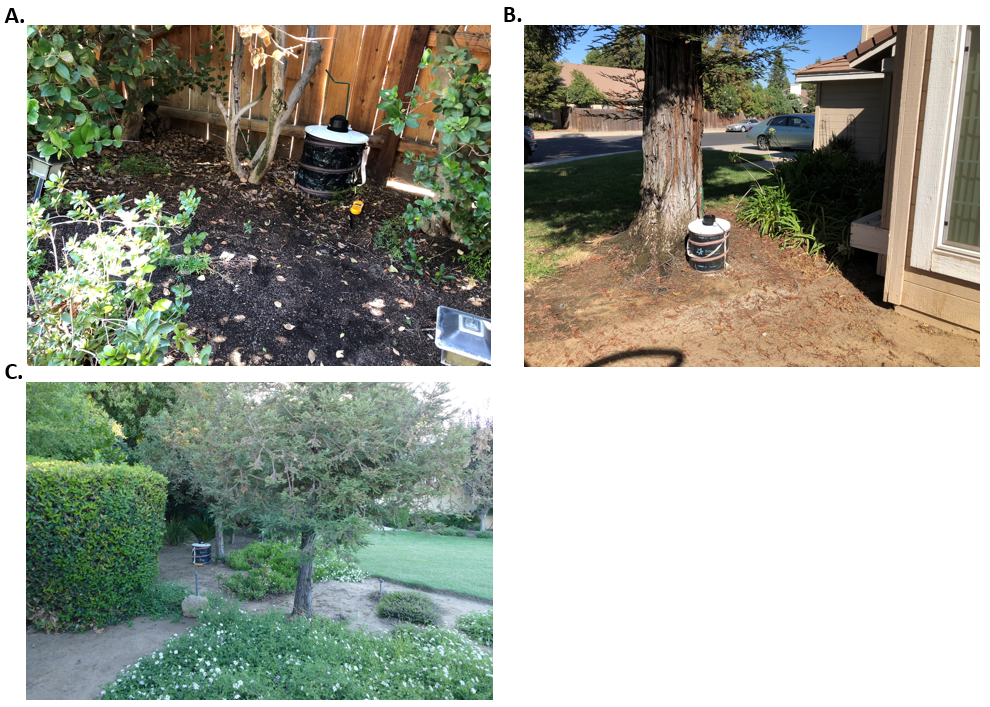

Supplement: S4 Fig — BGS trap proximity to wall or fence A. <1 m, B. 1–2 m and C. >2 m. (TIF) [file pntd.0008367.s005.tif]

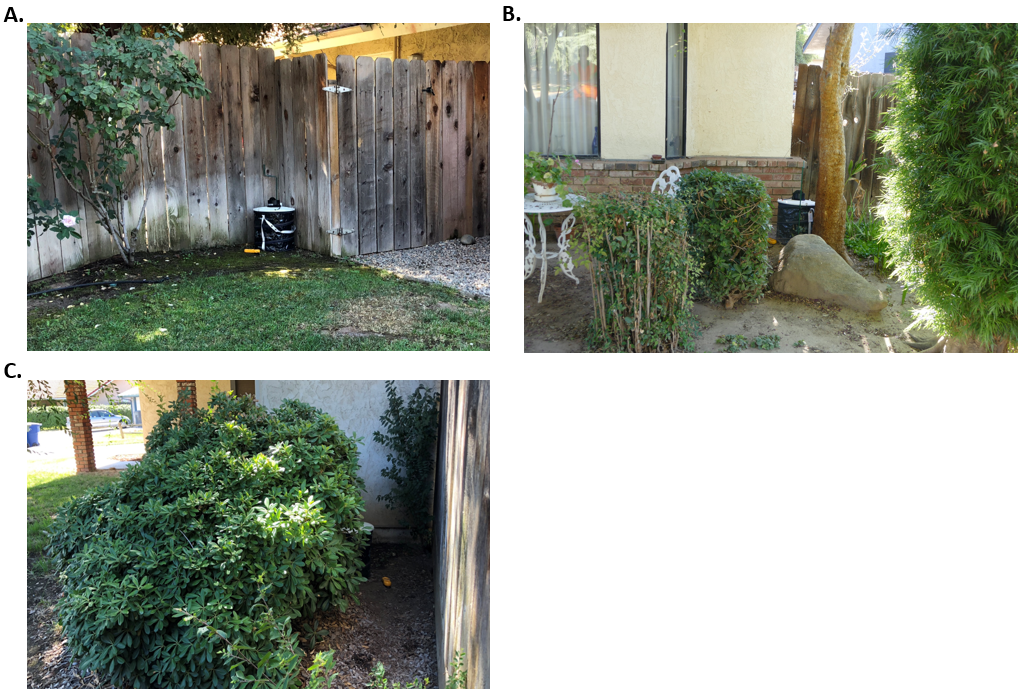

Supplement: S5 Fig — Visual competition to the BGS trap A. low, B. medium and C. high. (TIF) [file pntd.0008367.s006.tif]

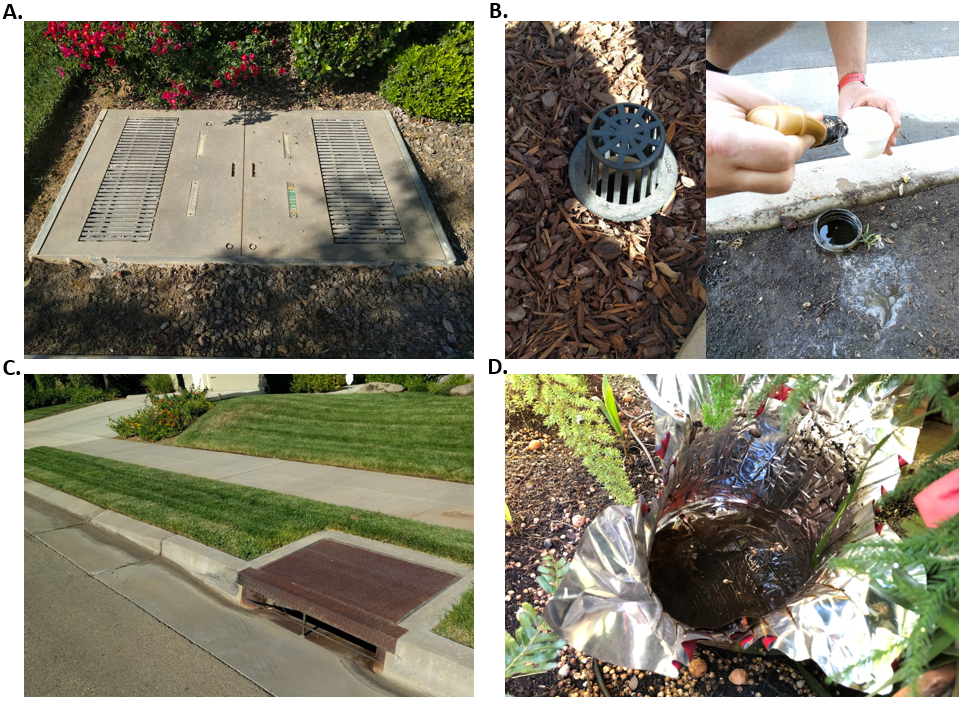

Supplement: S6 Fig — Potential larval habitats A. PG&E vault, B. yard drain, C. catch basin and D. yard container. (TIF) [file pntd.0008367.s007.tif]

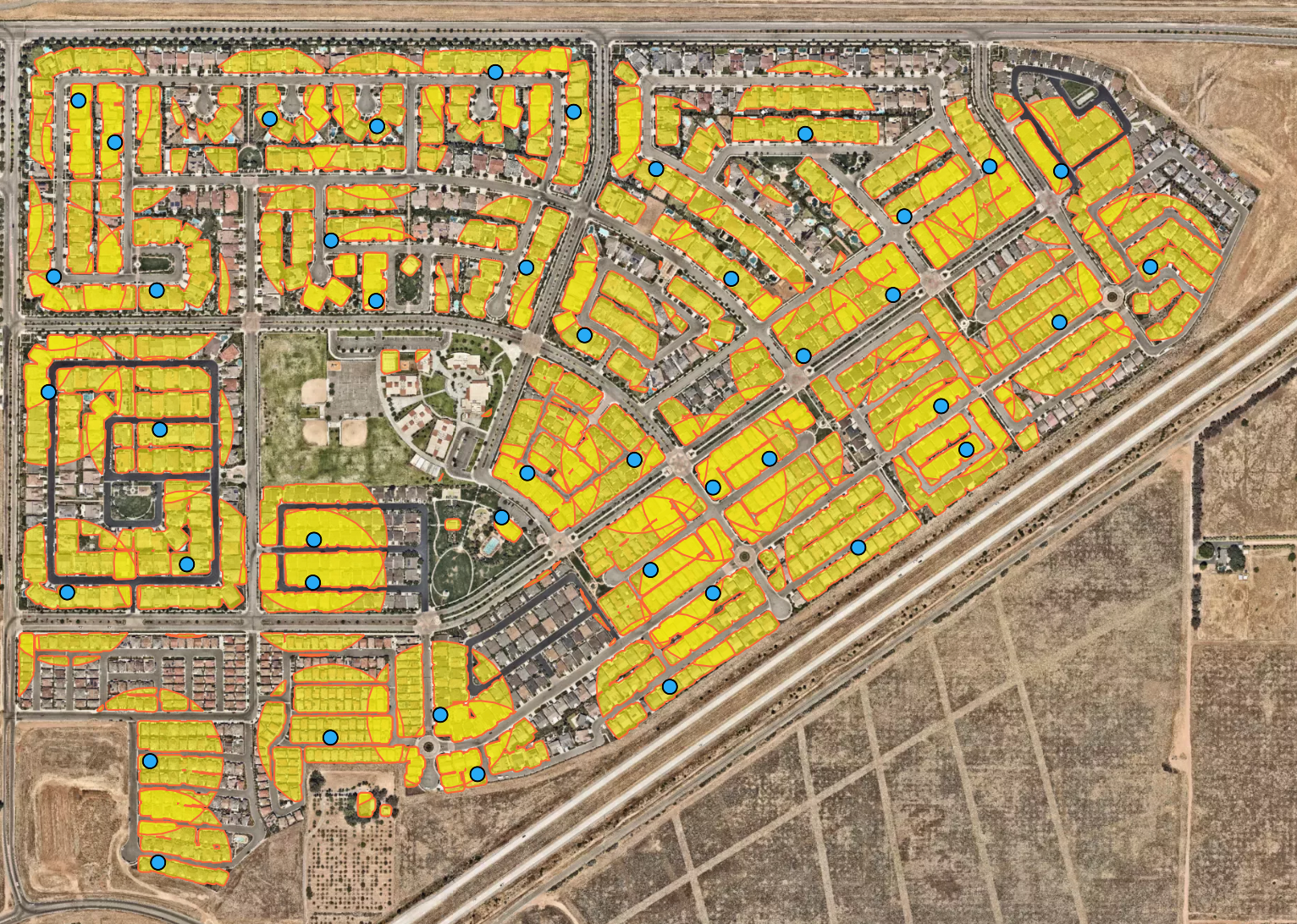

Supplement: S7 Fig — Residential habitat footprints (yellow areas) within 100 m radius of BGS traps (blue points) in T1, Fresno. Map created in QGIS version 3.4 with base layer sourced from The United States Geological Services (USGS). (TIF) [file pntd.0008367.s008.tif]

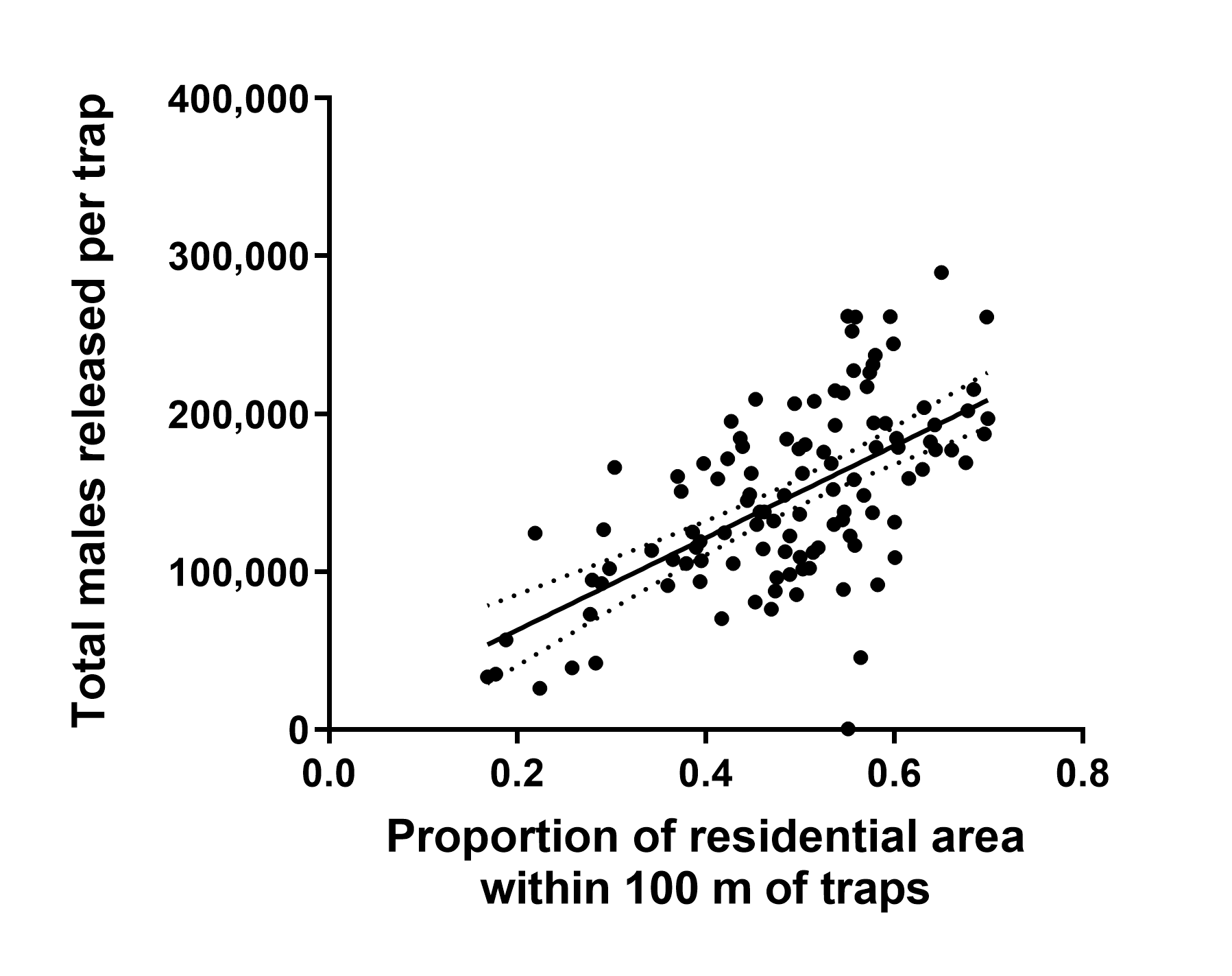

Supplement: S8 Fig — (TIF) [file pntd.0008367.s009.tif]
